# Study on health seeking behaviour and determinants of undiagnosed hypertension in poor households in the Philippines, part of the RESPOND study (SHARP-RESPOND)

**DOI:** 10.1371/journal.pgph.0004550

**Published:** 2025-05-28

**Authors:** Hau Man Harmony To, Benjamin Palafox, Dina Balabanova, Lia Palileo-Villanueva, Martin McKee

**Affiliations:** 1 Intensive Care Unit, Queen Elizabeth Hospital, King's Park, Hong Kong; 2 Centre for Global Chronic Conditions, London School of Hygiene & Tropical Medicine, London, United Kingdom; 3 College of Medicine, University of the Philippines Manila, Manila, Philippines; Erasmus University Rotterdam: Erasmus Universiteit Rotterdam, KINGDOM OF THE NETHERLANDS

## Abstract

Hypertension is one of the leading preventable causes of premature death. Although it can be effectively managed with relatively simple interventions, up to 50% of individuals with hypertension in low- and middle-income countries (LMICs) remain undiagnosed. Key factors influencing the health-seeking behaviour of patients with hypertension include household wealth, knowledge about hypertension, perceptions of treatment effectiveness, and access to blood pressure measurement. However, evidence on the facilitators and barriers to hypertension diagnosis in low-income households within LMICs remains inconsistent. This study aims to describe the characteristics and health-seeking behaviours of individuals with undiagnosed hypertension in low-income households in the Philippines and identify the factors influencing undiagnosed hypertension. The study included 516 people with hypertension from low-income households in the Philippines as part of the RESPOND study. Characteristics of participants with undiagnosed hypertension were compared to those with diagnosed hypertension to identify determinants of undiagnosed cases. A follow-up survey one year later gathered data on whether undiagnosed participants had subsequently received a formal diagnosis. In this study, 26.6% of people with hypertension in low-income households were undiagnosed. Over one year, only 25.4% of these undiagnosed individuals received a formal diagnosis. Factors associated with lower odds of undiagnosed hypertension included belief in the effectiveness of Western medicine, recent blood pressure measurement, receipt of health information in the preceding year, presence of comorbidities, and participation in social organisations. Conversely, living in rural areas, employment, and belief in the effectiveness of traditional medicine were linked to higher odds of remaining undiagnosed. A substantial proportion of people with hypertension in low-income households in the Philippines remain undiagnosed. Addressing this issue requires a multifaceted approach targeting the social determinants of health and addressing specific barriers to hypertension diagnosis. Insights from this study can inform strategies to improve hypertension control in other LMICs.

## Introduction

Cardiovascular diseases (CVDs) contribute to a growing global disease burden, rising from 10.8% of disability-adjusted life years (DALYs) lost in 1990 to 15.5% in 2019. [1] Much of this burden is preventable. Anti-hypertensive medications reduce cardiac and cerebrovascular complications, [2,3] yet only 30% of people with hypertension in low- and middle-income countries (LMICs) receive treatment, with less than 8% achieving control. [4] This is critical, as 80% of CVD-related deaths occur in LMICs [5].

Managing hypertension involves a dynamic process, from initial health system contact to diagnosis, treatment initiation, and long-term follow-up. [6,7] However, most studies on undiagnosed hypertension in LMICs are cross-sectional, [8–10] with limited longitudinal data on how health-seeking behaviours evolve or on factors influencing this change [7].

Additionally, much of the existing research relies on retrospective national surveys, needing more detailed information on healthcare costs, perceptions of hypertension, and attitudes toward Western versus traditional medicine. [11–13] A multi-year study in four middle-income countries found that only 30% of those with undiagnosed hypertension were diagnosed by follow up, and just 25% of untreated-diagnosed individuals began treatment. [7] This routine data analysis highlights gaps in understanding the barriers and facilitators to hypertension care.

Undiagnosed hypertension warrants attention due to its role in cardiovascular disease (CVD). Alone, and in combination with other risk factors such as hyperlipidaemia and smoking, it significantly increases the long-term risk of conditions like stroke and coronary heart disease. Measures that reduce these exposures reduce the substantial mortality, morbidity, and healthcare costs associated with CVD. Secondary prevention is less cost-effective than primary prevention, which requires early diagnosis and treatment of risk factors like hypertension. [3,14,15] Thus, understanding the health-seeking pathways followed by people with undiagnosed hypertension is crucial.

This study aims to explore the care trajectory of those with undiagnosed hypertension from low-income households in the Philippines and investigate key factors influencing their health-seeking behaviour. These factors include household wealth, [16,17] knowledge of hypertension [18–22], perception of treatment effectiveness [23,24] and having regular blood pressure measurements [6], all factors shown in various studies in LMIC to affect the health-seeking behaviours of adults with hypertension.

The starting point in the trajectory is diagnosis. This can be challenging because hypertension is often asymptomatic unless severe. A systematic review of methods of hypertension diagnosis in LMICs, which included 30 studies, emphasised the importance of routine blood pressure measurement during visits to primary care professionals. [6] Many studies, including two from Egypt and Malaysia, have shown how an absence of routine blood pressure measurement is associated with more patients being diagnosed late [21,25].

Once people are aware they have hypertension, the next steps are initiation of and adherence to treatment. A qualitative study within the RESPOND project, undertaken in the Philippines, revealed many misconceptions. Thus, hypertension was believed to be transient and there were ill-founded fears about long-term use of medication, leading to patient disengagement. [26] More generally, the absence of symptoms has been found, in countries as diverse as India, Malaysia, Colombia, Iran, and Kenya, to encourage a view that long-term treatment is not needed [18–21,27].

Even when established on treatment, patients may give it up if they believe that it is ineffective. This has been reported in, for example, Ghana and Nigeria, among those with doubts about the efficacy of Western medications. [23,24] In the RESPOND study, some participants viewed traditional medicine as an effective and accessible alternative, using herbal remedies as “drug holidays” once symptoms subsided, treating modern medications as optional [26].

## Aims and objectives

This study aims to provide a longitudinal analysis of the health-seeking behaviour of undiagnosed people with hypertension in the Philippines. It seeks to identify factors influencing undiagnosed hypertension and the determinants that lead to receiving a formal diagnosis within one year.

## Methods

### Ethics statement

Ethical approval for this study was granted by the Observational Research Ethics Committee at the London School of Hygiene & Tropical Medicine (Ref: 28510). Ethical approvals for the original RESPOND study were granted by the Observational Research Ethics Committee at the London School of Hygiene and Tropical Medicine and Research Ethics Boards at the University of the Philippines Manila (UPMREB-2017-481-01). All participants gave written informed consent.

#### Inclusion and exclusion criteria.

A total of 611 people with hypertension from low-income households in the Philippines participated in a prospective study under the RESPOND project. Further details are reported in a previously published protocol. [28] Briefly, the settings were seven urban communities in the City of Valenzuela in Metro Manila and 8 urban and 15 rural communities in Quezon province. A sampling frame was constructed using data on low-income households, defined as those qualifying for government subsidies under the 4P programme. [29] As reported previously, our sample was similar, in terms of median household income, levels of hypertension control, education and employment, with national data on this group. [30] After giving informed consent, individuals answered some screening questions to determine potential eligibility and had their blood pressure measured twice while in a sitting position after at least 5 minutes rest using an OMRON sphygmomanometer on the non-dominant arm. Participants met the following inclusion criteria: (1) aged 35–60 at the time of screening, (2) either self-reported history of diagnosed hypertension or were identified as hypertensive based on internationally accepted survey criteria, [31] which define hypertension as a blood pressure ≥140/90 mmHg, self-reported use of antihypertensive medication, or a diagnosis by a health professional, and (3) expected to remain at their current address for at least 18 months. Individuals with self-reported cancer or HIV were excluded, as their healthcare usage was deemed unrepresentative of the general adult population. 910 people were screened for inclusion.

#### Data collection.

A questionnaire using validated tools such as the Demographic and Health Surveys, [32] WHO STEPS, [33] World Values Survey, [34] and Living Conditions, Lifestyle and Health Survey [35,36] was administered at participants’ homes. Data on age, sex, education, marital status, household income, residence, and employment were collected. Participants (whether already diagnosed or not) were asked “In relation to the management of your blood pressure, who is your main point of care”, with choices of professional at hospital; professional at clinic; community health worker’; colleagues, friends or family; private physician; pharmacist; traditional healer; other; don’t know. They were then asked to pick up to two. All those reporting using a professional at hospital, professional at clinic, private physician, or pharmacist were recorded as having a regular provider. These variables were recorded at baseline and follow up. Household wealth was assessed using a validated asset-based wealth score using principal components analysis ranging from 0 to 1, with 1 indicating the highest wealth. [37] The first principal component from this analysis demonstrated good properties (i.e., high eigenvalue of 3.5 explaining 16% of total variation, with each component having sensible loadings), and the components of the wealth score and its parameters are described in S1 Table A. Healthcare expenditure derived from a combination of events in the previous month (consultation fees, one month supply of medications, diagnostic and lab tests, dental care, ambulance, other health care products or services) and the previous year (health insurance premiums, hospitalisations that required at least an overnight stay, long-term care facilities), are presented as the average spend in a month.

Participants reported their self-perceived knowledge of hypertension, while objective knowledge was assessed through five questions, forming a composite understanding score (S1 Table B). Data on comorbidities, perceived health status, and attitudes toward modern and traditional hypertension medications were collected. Routine blood pressure measurement practices were described. Social capital was assessed through perceived crisis support, participation in religious, cultural, and sports organisations, and trust in community and public institutions. Complete variable definitions are in S1 Table B.

Individuals without a prior diagnosis of hypertension but with blood pressure readings >140/90 mmHg were classified as having undiagnosed hypertension and were advised as such, and provided with information on the condition and how to seek medical advice from a government health facility. A follow-up interview one year later gathered information on their health-seeking behaviour, including healthcare system contact, whether a formal hypertension diagnosis was received, the type of facility and provider consulted, and adherence to prescribed medications. The baseline recruitment and survey were conducted between 15 May 2018 and 22 November 2018, and the follow-up between 3 July 2019 and 15 November 2019.

#### Analysis.

The socio-demographic characteristics of respondents, including age, sex, socioeconomic status, and area of residence, were described. Categorical variables were presented as percentages and continuous variables as means. Comparisons were made between (a) participants with undiagnosed hypertension and those with a history of diagnosed hypertension and (b) those with undiagnosed hypertension who received a diagnosis after one year versus those who remained undiagnosed. Categorical variables were analysed using chi-square tests and continuous variables with Wald tests.

Multivariate logistic regression identified determinants independently associated with (a) undiagnosed hypertension at baseline and (b) undiagnosed hypertension after one year. Potential determinants were selected based on the literature on barriers to chronic disease care in LMICs, including sex, [6] age, [38,39] highest level of education, [17,39,40] employment status, [26] household wealth, [16,17] knowledge about hypertension, [18,19,26] self-reported perception of the effectiveness of modern and traditional medicine as hypertension treatment, [23,24] and having a regular provider and routine blood pressure measurement practices. [21,26] Significant factors in univariate analyses were included multivariate models. Crude and adjusted odds ratios with 95% confidence intervals were reported (S1 Table C).

Longitudinal data after one-year follow-up detailed the health-seeking behaviour trajectories of diagnosed and undiagnosed individuals.

Summary statistics, crude and adjusted odds ratios from regression models, and Wald tests were weighted for sampling probability and community-level clustering to account for the sampling design. These adjustments ensure that the estimates represent low-income populations in the Philippines’ selected provinces and cities. Details on the calculation of the sampling weights can be found in S1 Table D.

All statistical analyses were conducted using IBM SPSS Statistics (Version 26)

## Results

### Included and missing data

**Fig 1:**
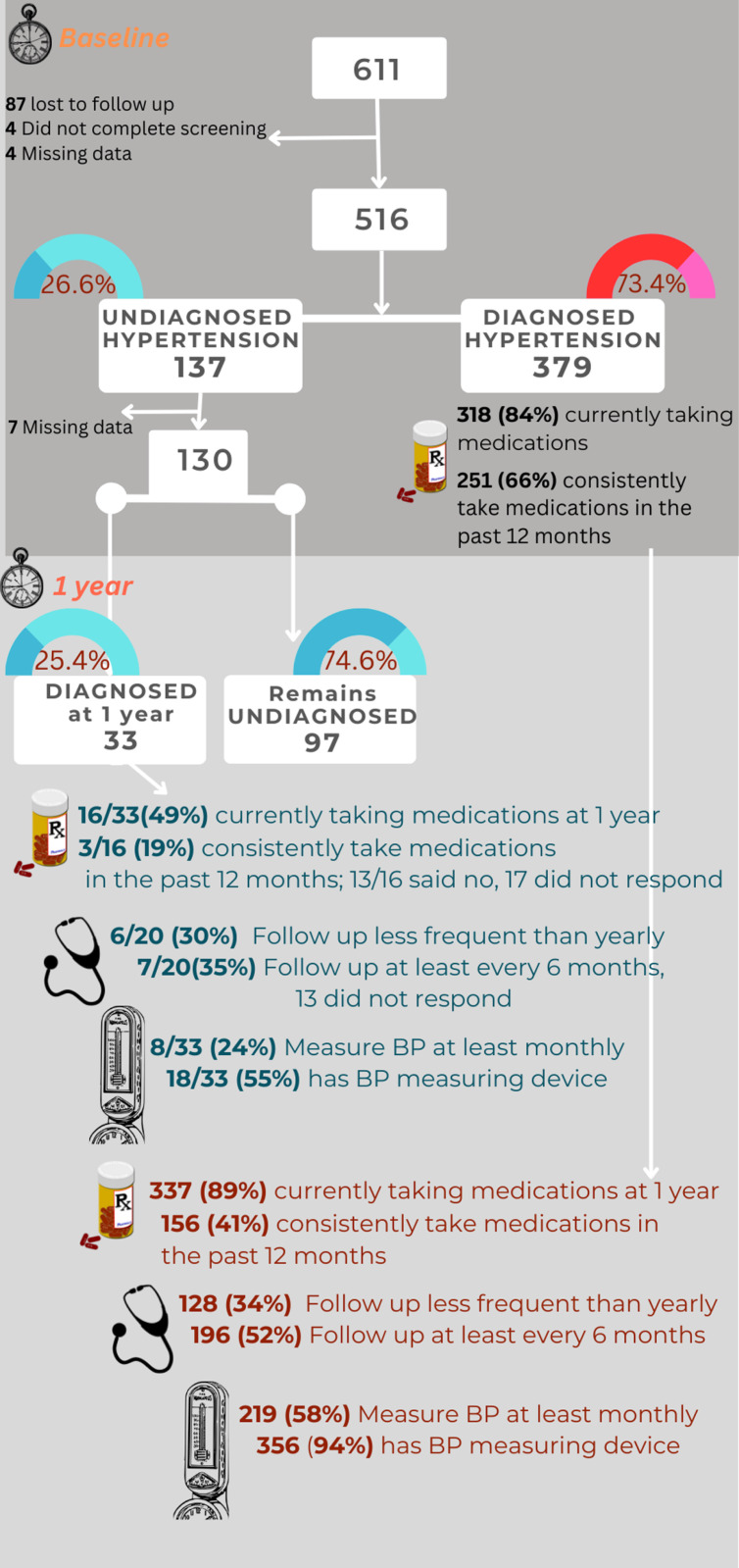
Flowchart showing patient flow and health-seeking behaviour.

A total of 611 low-income adults, either self-reported as hypertensive or identified as hypertensive during screening, were enrolled in the RESPOND study. Of these, 95 individuals were excluded (87 lost to follow-up at 1 year, four did not complete screening, and 4 had significant missing data). Excluded participants did not differ significantly from included participants regarding sex, residence (urban/rural), marital status, education, or asset-based wealth score. However, they were notably different in age, employment status, presence of comorbidities, and hypertension knowledge score (Table 1, Fig 1).

**Table 1:** Comparison between excluded and included data in baseline demographics, health status and knowledge of hypertension.

		Included (n = 516)	Excluded (n = 95)	p-value
**Sex**	Female	71.0% ± 0.7%	70.3% ± 0.4%	0.459
**Age**		54.97 ± 0.07	53.60 ± 0.15	**0.002**
**Residence**	Rural	11.8% ± 1.5%	10.4% ± 1.7%	0.492
**Marital status**	Married/cohabiting	74.0% ± 0.3%	74.6% ± 0.4%	0.415
**Education**	Post-secondary education	63.9% ± 0.7%	61.6% ± 0.5%	0.111
**Asset-based household wealth score (0 poorest - 1 wealthiest)**	mean ± SD	0.52 ± 0.00	0.53 ± 0.01	0.083
**Employment**	Employed	48.9% ± 0.4%	36.3% ± 0.6%	**<0.0001**
**Presence of NCD co-morbidity**		23.1% ± 0.4%	26.8% ± 0.5%	**<0.0001**
**Knowledge on Hypertension**	Score out of 5	3.10 ± 0.02	2.77 ± 0.01	**<0.0001**

Categorical variables are presented in proportion ± Standard error and compared with the Pearson chi-square test. Continuous variables are presented in mean ± standard error and compared with the Wald test. Analyses were weighted for sampling probability and adjusted for community-level clustering.

### Characteristics of undiagnosed hypertensive adults at baseline

Participants with undiagnosed hypertension were more likely to be working than not, and to be male rather than female (Table 2). They were slightly younger than those with diagnosed hypertension (52.13 ± 0.24 years vs. 55.75 ± 0.06 years, p < 0.0001) and more likely to live in rural areas (18.8% vs. 9.9%, p = 0.020). Their households spent less on healthcare both in absolute terms (138.64 ± 21.71 Pesos vs. 297.66 ± 6.19 Pesos, p = 0.002) and as a percentage of household expenditure (6.22% vs. 10.31%, p < 0.0001). There was no significant difference between the groups in terms of education level. In terms of health status, people with undiagnosed hypertension were less likely to have comorbid conditions (6.4% vs. 27.7%, p < 0.0001) and less likely to rate their health as poor (8% vs. 12.6%, p < 0.0001). They were less likely to have a regular healthcare provider (66.2% vs. 72.2%, p = 0.0006) and had received less health information in the past 12 months (27.5% vs. 41.1%, p = 0.001). Both groups had similar fatalistic views about health.

**Table 2:** Baseline demographics and characteristics of those with undiagnosed and diagnosed hypertension.

		Undiagnosed (n = 137)	Diagnosed (n = 379)	All (n = 516)	p-value
**Sex**	Female	59.2% ± 0.9%	74.3% ± 0.7%	71.0% ± 0.6%	**<0.0001**
**Age**		52.13 ± 0.24	55.75 ± 0.06	54.97 ± 0.07	**<0.0001**
**Residence**	Rural	18.8% ± 3.8%	9.9% ± 1.0%	11.8% ± 1.5%	**0.020**
**Marital Status**	married/cohabiting	77.4% ± 1.1%	73.0% ± 0.2%	74.0% ± 0.3%	**0.016**
**Post-secondary education**		66.8% ± 2.0%	63.1% ± 0.6%	63.9% ± 0.7%	0.108
**Currently employed**		60.2% ± 0.7%	45.8% ± 0.4%	48.9% ± 0.4%	**<0.0001**
**Household size**		4.24 ± 0.05	4.42 ± 0.01	4.38 ± 0.02	**0.014**
**Asset-based household wealth score(0–1, 1 wealthiest)**		0.51 ± 0.01	0.53 ± 0.00	0.52 ± 0.00	0.290
**Self-rated poor**		25.2% ± 0.7%	33.4% ± 0.4%	31.6% ± 0.3%	**0.001**
**Have regular healthcare provider**		66.2% ± 1.2%	72.2% ± 0.3%	71.0% ± 0.3%	**0.006**
**Health and health perception**					
**Self-rated to have poor health**		8% ± 0.5%	12.6% ± 0.3%	11.6% ± 0.3%	**<0.0001**
**Presence of non-communicable disease comorbidity**		6.4% ± 0.4%	27.7% ± 0.2%	23.1% ± 0.4%	**<0.0001**
**Blood pressure at first visit**	Systolic	157.04 ± 0.36	150.39 ± 0.17	151.83 ± 0.15	**<0.0001**
	Diastolic	99.68 ± 0.34	92.48 ± 0.11	94.04 ± 0.12	**<0.0001**
**Blood pressure at follow-up**	Systolic	150.70 ± 0.40	146.36 ± 0.31	147.30 ± 0.27	**0.001**
	Diastolic	96.07 ± 0.41	90.86 ± 0.21	91.98 ± 0.14	**0.001**
**Believes that Western medicines are effective**		60.9% ± 1.2%	88.9% ± 0.4%	82.8% ± 0.6%	**<0.0001**
**Believes that traditional medicines are effective**		63.5% ± 1.2%	53.2% ± 0.6%	55.4% ± 0.6%	**0.001**
**Regular blood pressure monitoring at least monthly**		35.7% ± 0.5%	73.1% ± 0.7%	65.0% ± 0.7%	**<0.0001**
**Knowledge on hypertension**					
**Self-rated to have poor knowledge of hypertension**		30.0% ± 1.3%	19.9% ± 0.6%	22.1% ± 0.7%	**0.001**
**Score on knowledge of hypertension(out of 5)**		2.95 ± 0.03	3.14 ± 0.01	3.10 ± 0.02	0.003
**Reception of any health information in the past 12 months**		27.5% ± 1.6%	41.1% ± 0.8%	38.2% ± 0.8%	**0.001**
**Social participation and confidence in institutions**					
**An active member of sports or recreational organisations**		5.1% ± 0.9%	1.5% ± 0.2%	2.3% ± 0.3%	**0.004**
**An active member of art, music or educational organisations**		1.1% ± 0.5%	2.3% ± 0.2%	2.0% ± 0.2%	0.161
**An active member of humanitarian or charitable organisations**		17.1% ± 1.1%	25.1% ± 0.3%	23.3% ± 0.4%	**0.002**
**An active member of church/religious organisations**		26.2% ± 1.0%	41.8% ± 0.4%	38.4% ± 0.3%	**<0.0001**
**An active member of any of the above organisations**		36.0% ± 1.2%	51.3% ± 0.3%	48.0% ± 0.3%	**<0.0001**
**Score for trust in personal relationships(out of 4)**		1.53 ± 0.12	1.69 ± 0.06	1.66 ± 0.07	0.056
**Have trust in the health system**		86.7% ± 0.2%	90.3% ± 0.2%	89.5% ± 0.2%	**<0.0001**
**Score for trust in public institutions (out of 4)**		2.41 ± 0.03	2.58 ± 0.01	2.54 ± 0.01	**0.006**

Categorical variables are presented in proportion ± Standard error and compared with the Pearson chi-square test. Continuous variables are presented in mean ± standard error and compared with the Wald test. Analyses were weighted for sampling probability and adjusted for community-level clustering.

Regarding hypertension knowledge, undiagnosed participants were more likely to rate their knowledge as low (30.0% vs. 19.9%, p = 0.001) and had lower objective knowledge scores (2.95 vs. 3.14, p = 0.003). They were also more likely to believe misconceptions, such as stopping medication when blood pressure is normal (48.6% vs. 41.9%, p < 0.0001) or taking medication only when feeling unwell (41.9% vs. 29.4%, p = 0.001).

Regarding the perception of treatment effectiveness, participants with undiagnosed hypertension were less likely to believe in the effectiveness of Western medications (60.9% vs. 88.9%, p < 0.0001) and more likely to believe in the effectiveness of traditional treatments (63.5% vs. 53.2%, p = 0.001) compared to those with diagnosed hypertension. The proportion of participants with blood pressure measured at least monthly was much lower among those with undiagnosed hypertension (35.7% vs. 73.1%, p < 0.0001).

Regarding social cohesion, those with undiagnosed hypertension were less likely to trust people in their neighbourhood (42.3% vs. 52.0%, p = 0.005). They were less active in organisations like sports, arts, or religious groups (36.0% vs. 51.3%, p < 0.0001). They also had lower trust in public institutions (2.41 vs. 2.58, p = 0.006) and were less likely to trust the health system (86.7% vs. 90.3%, p < 0.0001). Further details on the characteristics of the groups at baseline and follow up are provided in S1 Table C.

### Correlates of being undiagnosed among hypertensive adults at baseline

Table 3 shows the findings of the multivariate analysis of those variables showing a significant association with having undiagnosed hypertension in univariate analyses. Participants from rural areas had twice the odds of undiagnosed hypertension (aOR 2.36, 95% CI 1.48-3.76). Employment was also linked to higher odds of undiagnosed hypertension (aOR 1.57, 95% CI 1.38-1.79).

**Table 3:** Multivariate logistic regression model for undiagnosed hypertension.

		Crude Odds Ratio (OR)	95% CI	p-value	Adjusted OR	95% CI	p-value
**Sex**	Female	1(reference)			1(reference)		
	Male	1.98	1.69-2.34	**<0.0001**	1.12	0.87-1.44	0.292
**Age**		0.96	0.95-0.97	**<0.0001**	0.96	0.95-0.97	**<0.0001**
**Residence**	Urban	1(reference)			1(reference)		
	rural	2.12	1.20-3.73	**0.021**	2.36	1.48-3.76	**0.007**
**Post-secondary education**	No	1(reference)			1(reference)		
	Yes	1.18	0.94-1.47	0.108	0.91	0.82-1.02	0.082
**Employment**	Unemployed	1(reference)			1(reference)		
	Employed	1.79	1.67-1.93	**<0.0001**	1.57	1.38-1.79	**0.001**
**Asset-based wealth score**		0.64	0.23-1.75	0.281	2.2	0.89-5.45	0.074
**Presence of NCD comorbidity**	No	1(reference)			1(reference)		
	Yes	0.18	0.15-0.21	**<0.0001**	0.33	0.27-0.39	**<0.0001**
**Regular healthcare provider**	No	1(reference)			1(reference)		
	Yes	0.75	0.65-0.88	**0.006**	0.99	0.83-1.19	0.913
**Knowledge score on hypertension**		0.86	0.81-0.92	**0.003**	1.02	0.99-1.04	0.132
**Received any health information in the past 12 months**	know more than name	1(reference)			1(reference)		
	Know nothing or only the name	0.54	0.45-0.66	**0.001**	0.64	0.53-0.78	**0.003**
**Perception of Western medicine**	Neutral or ineffective	1(reference)			1(reference)		
	Effective	0.2	0.18-0.21	**<0.0001**	0.17	0.15-0.19	**<0.0001**
**Perception of traditional medicine**	Neutral or ineffective	1(reference)			1(reference)		
	Effective	1.53	1.34-1.74	**0.001**	1.43	1.13-1.80	**0.013**
**Routine measure of BP at least monthly**	No	1(reference)			1(reference)		
	Yes	0.20	0.19-0.23	**<0.0001**	0.29	0.24-0.35	**<0.0001**
**An active member of social organisations**	No	1(reference)			1(reference)		
	Yes	0.53	0.47-0.61	**<0.0001**	0.64	0.57-0.71	**<0.0001**
**Have trust in the health system**	No	1(reference)			1(reference)		
	Yes	0.7	0.66-0.74	**<0.0001**	0.80	0.62-1.02	0.063

Having an NCD co-morbidity was associated with almost 3 times lower odds of undiagnosed hypertension (aOR 0.33, 95% CI 0.27-0.39). Other protective factors included receiving health information in the past 12 months (aOR 0.64, 95% CI 0.53-0.78), monthly blood pressure measurement (aOR 0.29, 95% CI 0.24-0.35), and being an active member of social organisations (aOR 0.64, 95% CI 0.57-0.71).

Belief in the effectiveness of Western medicine was associated with 5 times lower odds of undiagnosed hypertension (aOR 0.17, 95% CI 0.15-0.19), while belief in traditional medicine was linked to higher odds (aOR 1.43, 95% CI 1.13-1.80).

Household wealth, knowledge of hypertension, having a regular provider, and trust in the health system were no longer associated with undiagnosed hypertension in the multivariable model. In the case of household wealth, inspection of the data suggested a non-linear association between wealth and being diagnosed so, given the limited range of assets in this sample of poor people, the most likely explanation is that the univariate significance is a chance finding.

### Overview of health-seeking behaviour trajectory

Among the 516 participants included in the analysis, 137 (26.6%) had undiagnosed hypertension, while 379 (73.4%) reported a previous diagnosis (Fig 1). Of the 130 people with undiagnosed hypertension at baseline and who responded after 1 year follow up, 33 (25.4%) had received a formal diagnosis. While 16 of these 33 individuals (48.5%) reported current use of hypertension medication, only 3 out of 16 who responded to this question (18.8%) took their medication consistently in the past 12 months. Among the 379 people with previously diagnosed hypertension, 318 (83.8%) reported taking medication at baseline, with 251 of the 379 (66.2%) doing so consistently in the past 12 months. At 1 year, 337 (88.9%) of these 379 individuals were on medication, but only 156 (41.2%) adhered consistently in the previous 12 months.

### Health-seeking behaviour of those with undiagnosed hypertension at baseline and 1-year follow-up

At baseline, 61 of 137 people with undiagnosed hypertension (44.5%) received healthcare from clinics, 27 (19.7%) from family and friends, and 17 (12.4%) from hospitals. These were also the top sources of care for those with diagnosed hypertension. At 1-year follow-up, clinics provided care for the majority, 356 (93.9%) of 379 people with previously diagnosed hypertension and 28 of 33 (86.5%) people with newly diagnosed hypertension. Pharmacists at retail pharmacies were the second most common source of care, reported by 16 of 379 (4.2%) people with previously diagnosed and 5 of 33 (15.2%) with newly diagnosed hypertension (S1 Table C).

About one-third of the 379 with previously diagnosed (n = 128, 33.8%) and 33 with newly diagnosed hypertension (n = 6 out of 20 who responded to this question, 30.0%) saw their providers less than once a year at 1 year follow up. Just over half of 379 with previously diagnosed (n = 196, 51.8%) and one-third of 33 with newly diagnosed hypertension (n = 7 out of 20 who responded to this question, 35%) were seen at least every 6 months at 1 year follow up.

A larger proportion of the 379 with previously diagnosed hypertension measured their blood pressure monthly (n = 219, 57.8%) than among the 33 who were newly diagnosed (n = 8, 24.2%). However, a high proportion of both groups owned sphygmomanometers: 94.4% of the previously diagnosed and 54.5% of those with newly diagnosed hypertension.

### Correlates of receiving formal diagnosis at 1-year follow-up among undiagnosed hypertensives

Univariate analysis comparing baseline demographics and health-seeking behaviours of people with newly diagnosed hypertension and those who remained undiagnosed (Table 4) showed no significant differences in age, urbanicity, asset-based wealth score, having a regular provider, or health information receipt in the past 12 months.

**Table 4:** Univariate comparison of those who received a formal diagnosis at 1-year follow-up with those who remained undiagnosed.

		No formal Diagnosis (n = 97)		Formally diagnosed at FU (n = 33)		p-value	
		Baseline	FU	Baseline	FU	Baseline	FU
**Sex**	Female	55.0% ± 1.0%	/	73.6% ± 2.0%	/	**0.002**	/
**Age**		51.45 ± 0.37	/	52.80 ± 0.36	/	0.067	/
**Residence**	Rural	20.9% ± 4.7%	/	16.5% ± 3.8%	/	0.258	/
**Marital Status**	married/ cohabiting	73.6% ± 1.7%	/	82.4% ± 1.4%	/	**0.032**	/
**Post-secondary education**		70.5% ± 2.5%	/	48.3% ± 1.9%	/	**0.001**	/
**Currently employed**		69.5% ± 0.6%	/	39.0% ± 2.9%	/	**0.001**	/
**Have regular healthcare provider**		64.9% ± 1.9%	/	69.8% ± 1.6%	/	0.167	/
**Blood pressure at first visit**	Systolic	158.44 ± 0.55	150.35 ± 0.63	155.11 ± 0.83	152.23 ± 1.04	**0.036**	0.226
	Diastolic	100.83 ± 0.46	96.14 ± 0.52	97.63 ± 0.48	97.05 ± 0.50	**0.013**	0.099
**Socioeconomic status**							
**Asset-based household wealth score (0–1, 1 wealthiest)**		0.50 ± 0.01	/	0.51 ± 0.01	/	0.369	/
**Total monthly household expenditure per capita, PH Pesos**		2099.76 ± 93.07	/	1201.03 ± 58.40	/	**<0.0001**	/
**Logged monthly household health expenditure per capita, PH Pesos**		7.99 ± 0.03	/	7.50 ± 0.08	/	**0.005**	/
**Total monthly household HEALTH expenditure per capita**		117.90 ± 7.96	/	72.68 ± 2.42	/	**0.001**	/
**Health expenditure as a percentage of total household expenditure per month**		5.58 ± 0.14	/	7.38 ± 0.31	/	**<0.0001**	/
**Household size**		4.27 ± 0.09	/	4.37 ± 0.02	/	0.389	/
**Self-rated poor**		25.0% ± 1.5%	/	32.8% ± 1.2%	/	**0.031**	/
**Health and health perception**							
**Self-rated to have poor health**		9.2% ± 0.6%	/	7.0% ± 0.9%	/	0.117	/
**Presence of non-communicable disease comorbidity**		9.3% ± 0.8%	/	0(0.0%)	/	**0.002**	/
**Receipt of any health information in the past 12 months**		25.1% ± 1.7%	/	28.7% ± 2.7%	/	0.224	/
**All things considered, how satisfied are you with your life?**		6.40 ± 0.26	7.11 ± 9.76	5.31 ± 0.10	6.28 ± 2.28	**<0.0001**	**0.034**
**Do you feel they have completely free choice and control over your life?**		6.36 ± 0.20	7.20 ± 9.72	5.99 ± 0.08	5.84 ± 1.99	**<0.0001**	**0.019**
**Knowledge and perception of hypertension**							
**Self-rated to have poor knowledge on hypertenion**		33.4% ± 1.7%	7.3% ± 1.3%	17.2% ± 1.3%	8.2% ± 1.0%	**0.001**	0.634
**Score on knowledge on hypertension(out of 5)**		2.97 ± 0.04	3.04 ± 0.05	3.02 ± 0.04	3.11 ± 0.06	**0.002**	**0.040**
**Believes that western medicines are effective**		58.6% ± 1.3%	61.8% ± 2.4%	62.1% ± 1.2%	83.5% ± 1.9%	**0.001**	**0.002**
**Believes that traditional medicines are effective**		67.7% ± 1.1%	56.7% ± 1.3%	56.1% ± 2.3%	59.8% ± 2.6%	**0.002**	0.318
**Regular blood pressure monitoring at least monthly**		33.4% ± 0.6%	15.1% ± 1.5%	31.7% ± 0.9%	38.1% ± 2.6%	0.149	**0.002**
**Social participation and confidence in institutions**							
**Active member of sports or recreational organizations**		4.6% ± 1.0%	3.6% ± 0.8%	7.8% ± 0.8%	7.9% ± 0.7%	**0.034**	**0.034**
**Active member of humanitarian or charitable organizations**		14.4% ± 1.4%	25.9% ± 1.6%	16.6% ± 1.6%	13.1% ± 2.4%	0.189	**0.024**
**Active member of church/religious organizations**		27.7% ± 1.1%	36.3% ± 0.6%	17.0% ± 1.1%	34.7% ± 2.2%	**<0.0001**	0.491
**Active member of any social organization above**		33.7% ± 1.3%	49.8% ± 0.9%	32.8% ± 1.5%	36.0% ± 1.8%	0.346	**0.003**
**Trust score personal (Out of 5)**		1.52 ± 0.13	2.06 ± 0.03	1.50 ± 0.11	2.07 ± 0.03	0.651	0.800
**Have trust in the health system**		87.6% ± 0.7%	72.8% ± 1.7%	80.7% ± 1.7%	83.2% ± 2.2%	**0.037**	**0.007**
**Score for trust for public institutions (Out of 4)**		2.34 ± 0.03	2.58 ± 1.54	2.54 ± 0.06	2.24 ± 1.66	**0.044**	0.080
**Have easy access to blood pressure measuring device**		/	78.0% ± 4.2%	/	82.7% ± 0.9%	/	0.316

Categorical variables are presented as proportion ± Standard error and compared with Pearson chi-square test. Continuous variables are presented as mean ± standard error and compared using a Wald test. Analyses were weighted for sampling probability and adjusted for community-level clustering.

Those who remained undiagnosed at 1 year were more likely to be working and more likely to be male. They were also less likely to believe in the effectiveness of Western medications and more likely to believe in traditional medicine for hypertension treatment.

Factors associated with lower odds of remaining undiagnosed at 1 year among those with undiagnosed hypertension at baseline included belief in the effectiveness of Western medicine (aOR 0.51, 95% CI 0.43-0.60) and regular blood pressure measurement at least monthly (aOR 0.26, 95% CI 0.13-0.56) (Table 5). Notably, these factors were also linked to lower odds of undiagnosed hypertension at baseline.

**Table 5:** Multivariate logistic regression of remaining undiagnosed at follow-up among patient with undiagnosed hypertension (n = 130).

		Crude Odds ratio (n = 137)	95% CI	p value	Adjusted odds ratio* (n = 137)	95% CI	p value
**Sex**	Female	1(reference)			1(reference)		
	Male	2.28	1.63-3.18	**0.002**	1.34	0.68-2.63	0.389
**Age**		0.99	0.97-1.00	0.063	1.03	1.01-1.05	**0.007**
**Residence**	Urban	1(reference)			1(reference)		
	rural	1.34	0.72-2.50	0.259	0.91	0.32-2.59	0.808
**Post-secondary education**	No post sec education	1(reference)			1(reference)		
	Post sec education	2.56	1.95-3.37	**0.001**	4.22	2.69-6.62	**0.001**
**Employment**	Unemployed	1(reference)			1(reference)		
	Employed	3.57	2.50-5.08	**0.001**	3.35	1.97-5.70	**0.003**
**Asset-based wealth score**		0.78	0.40-1.55	0.377	0.88	0.88-1.78	0.389
**Knowledge score on hypertension**		1.16	1.09-1.24	**0.002**	1.23	1.00-1.53	**0.052**
**Perception of western medication**	Neutral or ineffective	1(reference)			1(reference)		
	Effective	0.87	0.83-0.90	**0.001**	0.51	0.43-0.60	**<0.0001**
**Perception of traditional medication**	Neutral or ineffective	1(reference)			1(reference)		
	Effective	1.64	1.36-1.98	**0.002**	3.34	2.05-5.44	**0.002**
**Trust in health system**	No	1(reference)			1(reference)		
	Yes	0.54	0.39-0.76	**0.007**	0.73	0.45-1.16	0.133
**Regularly BP measure at least monthly**	No	1(reference)			1(reference)		
	Yes	0.29	0.18-0.47	**0.002**	0.26	0.13-0.56	**0.008**

Adjusted odds ratios presented were mutually adjusted for other co-variates listed. Analyses were weighted for sampling probability and adjusted for community-level cluster.

Factors associated with higher odds of remaining undiagnosed at 1 year included post-secondary education (aOR 4.22, 95% CI 2.69-6.62), employment (aOR 3.35, 95% CI 1.97-5.70), and belief in the effectiveness of traditional medicine (aOR 3.34, 95% CI 2.05-5.44). Employment and belief in effectiveness of traditional medicine were also associated with higher odds of undiagnosed hypertension at baseline.

## Discussion and recommendations

Four key factors influencing the health-seeking behaviour of patients with hypertension in LMICs were identified in the literature, primarily focusing on medicine adherence. Our study aimed to examine these factors’ impact on the diagnosis stage of hypertension among low-income individuals in the Philippines and identify other important determinants at this stage. Additionally, it provides a longitudinal overview over time of health-seeking behaviour among those with undiagnosed hypertension. This study has limitations. The sample size is relatively small, limiting the robustness of findings regarding determinants of diagnosis over time. The small number of diagnosed individuals also restricted our understanding of medical adherence and reasons for non-adherence. The 1-year follow-up focused mainly on diagnosis and treatment initiation but was too short to assess retention in the health system over the medium to long term. Nonetheless, this cohort provides new insights into the health-seeking trajectory of low-income patients with hypertension in the Philippines. It highlights key determinants of diagnosis, an essential step in improving hypertensive care and preventing long-term complications. Another limitation is that hypertension was defined according to internationally agreed standards for population surveys, [31] which may differ from clinical diagnostic criteria requiring repeated blood pressure measurements in specific settings.

Our study found that perception of treatment effectiveness (both Western and traditional) and regular BP measurement were significant correlates of undiagnosed hypertension, while household wealth and hypertension knowledge were not. In addition, we identified three factors associated with lower odds of undiagnosed hypertension: receipt of health information in the past 12 months, presence of an NCD comorbidity, and active participation in social organisations. Conversely, two factors associated with higher odds of undiagnosed hypertension were employment, and rural residence. Understanding these factors can inform targeted policy changes. Each factor is discussed, including our cohort’s findings, existing literature, and recommendations for policy translation.

### Summary of health-seeking trajectories

Out of 516 adults with hypertension in this study, 26.6% were undiagnosed, and only 25.4% of those with undiagnosed hypertension received a diagnosis over one year. This finding aligns with a recent survey across four LMICs, where only 30% of those with undiagnosed hypertension were diagnosed after 5–9 years of follow up. Among those diagnosed, 20.1% were not on medication in that study (7), similar to our finding, where 83.8% of diagnosed participants were on medication. The proportion of people with undiagnosed hypertension in our cohort was similar in rural Ethiopia (21.3%), [8] but lower than in Indonesia (56.2%), [16] and Malaysia (51.6%), [10] which is counterintuitive as the latter two studies were nationally representative rather than limited to poor households.

### Household wealth

In our cohort, household wealth did not significantly differ between those with undiagnosed and diagnosed hypertension. Previous studies in Indonesia and Kenya identified disposable income as a facilitator for diagnosis, where out-of-pocket costs were a barrier. [16,17] In contrast, Filipinos have access to free public primary healthcare, including hypertension consultation and medication, which may explain the difference in our cohort.

### Knowledge about hypertension

Our cohort found that undiagnosed participants were more likely to report poor knowledge of hypertension and scored lower on an objective knowledge test. This aligns with studies in Kenya, Belize, and Ethiopia, [8,17,40] where poor understanding of hypertension hindered care-seeking; however, knowledge score was not a significant determinant of diagnosis in our study. This suggests that, while knowledge is crucial for recognising the need for medical care, factors like treatment perception and healthcare access may play a larger role in whether knowledge leads to action.

### Perception of the effectiveness of Western and traditional medicine

Perception of the effectiveness of Western and traditional medicine for hypertension treatment had contrasting effects on diagnosis in our cohort. Believing that Western medicine was effective reduced the odds of undiagnosed hypertension by 5 times and the odds of remaining undiagnosed after one year by half (Tables 3 and 5). Conversely, belief in traditional medicine increased the odds of undiagnosed hypertension at baseline by 1.4 times and the odds of remaining undiagnosed after one year by 3.3 times.

These findings align with existing literature. A study from Ghana found that negative perceptions of mainstream medications led to treatment interruption. [23] In Ethiopia, a favourable attitude toward prescription medications increased adherence almost 10 times. [41] The perception of Western medicine’s effectiveness likely encourages seeking a formal diagnosis, while belief in traditional medicine may divert patients from diagnosis, as it can be accessed without formal healthcare. An added advantage of traditional medicine documented in the digital diaries of respondents from the same study was that herbal medicine could be home-grown without needing a clinical visit. [26] Studies from Kenya show that traditional healers can lead patients away from the formal health system, [17,20] and a qualitative study on the RESPOND data indicated that participants often preferred herbal medicine over prescriptions. [42] Quantitative analysis from the RESPOND dataset showed that belief in traditional and complementary medicine (TCAM) was associated with a higher likelihood of using TCAM concurrently with prescription medication in the Philippines and Malaysia. [43] National data from Malaysia showed that 60% of TCAM users used it as an alternative to prescription medicine, while 41% used it alongside it [42].

While evidence on the benefits or harms of traditional medicine for hypertension is mixed, there is strong evidence that TCAM use reduces adherence to prescription medications. [44] Our study shows that the perception of traditional medicine plays a significant role in both the diagnosis stage and the follow-up care for hypertension.

### Regular blood pressure measurement at least monthly

In our cohort, we found no significant association between having a regular healthcare provider and formal diagnosis. However, most participants did have one, and it is likely that our study was underpowered to detect any difference. Monthly blood pressure measurement, which was more common among participants with diagnosed hypertension than those with undiagnosed hypertension (73.1% ± 0.7% vs. 35.7% ± 0.5%, p < 0.0001), was also independently associated with 3 times lower odds of undiagnosed hypertension at baseline and nearly 4 times lower odds of remaining undiagnosed at 1-year follow-up (Table 3).

The importance of regular blood pressure measurement is supported by literature, with primary care visits being a key facilitator of diagnosis. The asymptomatic nature of hypertension until it becomes severe, along with non-specific symptoms, explains the need for regular monitoring. A study in Sri Lanka found that most patients discovered their hypertension incidentally during unrelated healthcare visits [45].

Would population-wide screening improve hypertension diagnosis? Experience from a Tanzania screening program found low follow-up rates, with only 34% of identified hypertensives attending healthcare providers within a year and 5% starting on medication. [38] Similarly, a hypertension screening program in Malaysia showed low follow-up rates [21].

Barriers to healthcare linkage following screening in these studies included lack of knowledge, competing priorities like work, and reliance on ineffective “wonder drugs” and herbs with no proven effectiveness. [21,38] Some of these same barriers, including employment, were also linked to undiagnosed hypertension in our cohort, as discussed further below.

An important question is whether low-income households in the Philippines would benefit from a screening program. In our cohort, around 80% of those with undiagnosed hypertension reported easy access to a blood pressure measuring device (82.7% of those diagnosed at 1 year and 78.0% of those who remained undiagnosed). This suggests that lack of access to blood pressure measurement is not a significant barrier to diagnosis, but rather to all the other elements that are required for a comprehensive response, including access to affordable treatment, effective follow up, and peer support.

### Employment and living in rural areas as a barrier to hypertension diagnosis

Employment was identified as a determinant of undiagnosed hypertension, with 1.5 times higher odds of being undiagnosed at baseline and 3.4 times odds of remaining undiagnosed at 1-year follow-up in our cohort.

The relationship between work and health-seeking behaviour has been explored in multiple studies. In Iran, busy work was linked to poorer medicine adherence. [27] A study in Namibia found 60% of missed medical appointments were due to work commitments. [46] Similarly, research in Ghana revealed that sustaining livelihoods led to the de-prioritization of medical treatment. [23] A qualitative analysis of RESPOND data from low-income households in the Philippines indicated that participants prioritised work over medical check-ups, especially when waiting times or clinic hours clashed with work schedules, particularly in informal sectors. [26] In Malaysia, participants described challenges such as private companies not accepting “time off” while doctors could not provide medical leave for short clinic visits. [21] Institutional barriers often deter hypertensive patients from seeking care.

Improving health facility accessibility may also be beneficial. Expensive transport costs were a significant barrier to seeking care after positive screening in a community program in Uganda. [39] Travel distance and convenience were central to healthcare access for RESPOND participants in the Philippines. Urban participants benefited from more facility options, while rural participants in Quezon Province had limited access, relying on public facilities that sometimes ran out of free hypertensive medications. Reaching private providers required two hours of travel. [26] This difficulty may explain why living in rural areas was associated with twice the odds of undiagnosed hypertension (Table 3).

Reimbursing transport costs in Haiti increased clinic visits for anti-retroviral treatment. [47] In Liberia, a program addressing healthcare barriers through transport reimbursements, food support, and social assistance improved tuberculosis treatment coverage from 7.7% to 43.2%. [48] While infectious disease and hypertension treatments differ, this targeted intervention approach is appropriate.

### NCD comorbidity, active participation in social organisations and receipt of health information in the preceding 12 months

Our study found that having an NCD co-morbidity was associated with one-third lower odds of undiagnosed hypertension (Table 3). Few studies have examined this relationship. A systematic review of hypertension treatment in LMICs found inconclusive evidence regarding co-morbidities and medicine adherence. [6] One explanation for our finding is that patients with co-morbidities are more likely to see healthcare providers who may screen for hypertension during treatment.

Participants active in social organisations had nearly half the odds of undiagnosed hypertension (Table 3). While social networks are known to support long-term health retention and medication adherence for hypertensives, [6] their role in diagnosis is less understood. Social organisation participation may reduce undiagnosed hypertension by spreading health information. Supporting this, receiving health information in the past year was linked to nearly half the odds of undiagnosed hypertension (Table 3).

## Recommendations

An appropriate response to these findings requires a multi-faceted response. Health professionals can raise awareness of the silent nature of hypertension and the importance of regular check-ups while dispelling fears about long-term medication. Community-driven initiatives, such as peer-led education and leveraging social networks, may further encourage diagnosis and adherence. Routine blood pressure screening must be more accessible, with incentives for regular check-ups, especially for workers who struggle to prioritise healthcare visits. Expanding mobile clinics and telemedicine services can bridge gaps for rural populations, while flexible clinic hours and employer support can help working individuals access care. Building trust in Western medicine is crucial. Health professionals should actively address concerns about antihypertensive drugs, while also engaging traditional healers to promote safe, evidence-based treatments. Health systems must ensure consistent medication availability and streamline access by reducing clinic wait times and offering transport reimbursements where needed.

## Conclusion

Among people with hypertension from low-income households in the Philippines, 26.6% were undiagnosed, and only 25.4% of these individuals received a diagnosis within a year. Factors associated with lower odds of undiagnosed hypertension included trust in Western medicine, monthly blood pressure checks, recent receipt of health information, presence of an NCD comorbidity, and participation in social organisations, while rural residence, employment, and belief in traditional medicine were linked to higher odds. Addressing these barriers through a multi-faceted approach that builds confidence in mainstream healthcare and tackles social determinants of health requires robust intersectoral policies, offering lessons for improving hypertension control in other LMICs.

## Supporting information

S1 Tables Table A: List of household assets/characteristics used in the construction of the household wealth index using principal components analysis (mean and standard deviation). Table B: Variable definition. Table C: Medical adherence and health seeking behaviour of previously known, newly diagnosed and undiagnosed hypertensives.Table D: Derivation of the probability-based sampling weights. (DOCX)

## References

[1] Institute for Health Metrics and Evaluation. GBD Compare 2023 [cited 2025 12th May]. Available from: https://vizhub.healthdata.org/gbd-compare/.

[2] Messerli FH, Williams B, Ritz E. Essential hypertension. Lancet. 2007;370(9587):591–603. doi: 10.1016/S0140-6736(07)61299-9 17707755

[3] Aminde LN, Takah NF, Zapata-Diomedi B, Veerman JL. Primary and secondary prevention interventions for cardiovascular disease in low-income and middle-income countries: a systematic review of economic evaluations. Cost Eff Resour Alloc. 2018;16:22. doi: 10.1186/s12962-018-0108-9 29983644 PMC6003072

[4] Mills KT, Bundy JD, Kelly TN, Reed JE, Kearney PM, Reynolds K, et al. Global Disparities of Hypertension Prevalence and Control: A Systematic Analysis of Population-Based Studies From 90 Countries. Circulation. 2016;134(6):441–50. doi: 10.1161/CIRCULATIONAHA.115.018912 27502908 PMC4979614

[5] World Health Organization. Global status report on noncommunicable diseases 2010 2011 [cited 2025 12th May]. Available from: https://apps.who.int/iris/handle/10665/44579

[6] Brathwaite R, Hutchinson E, McKee M, Palafox B, Balabanova D. The Long and Winding Road: A Systematic Literature Review Conceptualising Pathways for Hypertension Care and Control in Low- and Middle-Income Countries. Int J Health Policy Manag. 2022;11(3):257–68. doi: 10.34172/ijhpm.2020.105 32702800 PMC9278472

[7] Mauer N, Geldsetzer P, Manne-Goehler J, Davies JI, Stokes AC, McConnell M, et al. Longitudinal evidence on treatment discontinuation, adherence, and loss of hypertension control in four middle-income countries. Sci Transl Med. 2022;14(652):eabi9522. doi: 10.1126/scitranslmed.abi9522 35857627

[8] Gelassa FR, Birhanu A, Shibiru A, Nagari SL, Jabena DE. Undiagnosed status and associated factors of hypertension among adults living in rural of central, Ethiopia, 2020: Uncovering the hidden magnitude of hypertension. PLoS One. 2022;17(12):e0277709. doi: 10.1371/journal.pone.0277709 36520859 PMC9754235

[9] Hossain A, Suhel SA, Chowdhury SR, Islam S, Akther N, Dhor NR, et al. Hypertension and undiagnosed hypertension among Bangladeshi adults: Identifying prevalence and associated factors using a nationwide survey. Front Public Health. 2022;10:1066449. doi: 10.3389/fpubh.2022.1066449 36561867 PMC9763893

[10] Lim OW, Yong CC. The Risk Factors for Undiagnosed and Known Hypertension among Malaysians. Malays J Med Sci. 2019;26(5):98–112. doi: 10.21315/mjms2019.26.5.9 31728122 PMC6839659

[11] Ataklte F, Erqou S, Kaptoge S, Taye B, Echouffo-Tcheugui JB, Kengne AP. Burden of undiagnosed hypertension in sub-saharan Africa: a systematic review and meta-analysis. Hypertension. 2015;65(2):291–8. doi: 10.1161/HYPERTENSIONAHA.114.04394 25385758

[12] Palomo-Piñón S, Antonio-Villa NE, García-Cortés LR, Álvarez-Aguilar C, González-Palomo E, Bertadillo-Mendoza OM, et al. Prevalence and characterization of undiagnosed arterial hypertension in the eastern zone of Mexico. J Clin Hypertens (Greenwich). 2022;24(2):131–9. doi: 10.1111/jch.14414 34962058 PMC8845470

[13] Pengpid S, Peltzer K. Prevalence and associated factors of undiagnosed hypertension among adults in the Central African Republic. Sci Rep. 2022;12(1):19007. doi: 10.1038/s41598-022-23868-5 36347923 PMC9643345

[14] Becerra V, Gracia A, Desai K, Abogunrin S, Brand S, Chapman R, et al. Cost-effectiveness and public health benefit of secondary cardiovascular disease prevention from improved adherence using a polypill in the UK. BMJ Open. 2015;5(5):e007111. doi: 10.1136/bmjopen-2014-007111 25991449 PMC4452741

[15] Baigent C, Blackwell L, Collins R, Emberson J, Godwin J, et al.; Antithrombotic Trialists’ (ATT) Collaboration. Aspirin in the primary and secondary prevention of vascular disease: collaborative meta-analysis of individual participant data from randomised trials. Lancet. 2009;373(9678):1849–60. doi: 10.1016/S0140-6736(09)60503-1 19482214 PMC2715005

[16] Mahwati Y, Nurrika D, Latief K. The Determinants of Undiagnosed Hypertension Among Indonesian Adults: A Cross-sectional Study Based on the 2014-2015 Indonesia Family Life Survey. J Prev Med Public Health. 2022;55(1):60–7. doi: 10.3961/jpmph.21.500 35135049 PMC8841202

[17] Rachlis B, Naanyu V, Wachira J, Genberg B, Koech B, Kamene R, et al. Identifying common barriers and facilitators to linkage and retention in chronic disease care in western Kenya. BMC Public Health. 2016;16:741. doi: 10.1186/s12889-016-3462-6 27503191 PMC4977618

[18] Gabert R, Ng M, Sogarwal R, Bryant M, Deepu RV, McNellan CR, et al. Identifying gaps in the continuum of care for hypertension and diabetes in two Indian communities. BMC Health Serv Res. 2017;17(1):846. doi: 10.1186/s12913-017-2796-9 29282052 PMC5746011

[19] Legido-Quigley H, Camacho Lopez PA, Balabanova D, Perel P, Lopez-Jaramillo P, Nieuwlaat R, et al. Patients’ knowledge, attitudes, behaviour and health care experiences on the prevention, detection, management and control of hypertension in Colombia: a qualitative study. PLoS One. 2015;10(4):e0122112. doi: 10.1371/journal.pone.0122112 25909595 PMC4409332

[20] Naanyu V, Vedanthan R, Kamano JH, Rotich JK, Lagat KK, Kiptoo P, et al. Barriers Influencing Linkage to Hypertension Care in Kenya: Qualitative Analysis from the LARK Hypertension Study. J Gen Intern Med. 2016;31(3):304–14. doi: 10.1007/s11606-015-3566-1 26728782 PMC4762819

[21] Risso-Gill I, Balabanova D, Majid F, Ng KK, Yusoff K, Mustapha F, et al. Understanding the modifiable health systems barriers to hypertension management in Malaysia: a multi-method health systems appraisal approach. BMC Health Serv Res. 2015;15:254. doi: 10.1186/s12913-015-0916-y 26135302 PMC4489127

[22] Shima R, Farizah MH, Majid HA. A qualitative study on hypertensive care behavior in primary health care settings in Malaysia. Patient Prefer Adherence. 2014;8:1597–609. doi: 10.2147/PPA.S69680 25484577 PMC4240212

[23] Atinga RA, Yarney L, Gavu NM. Factors influencing long-term medication non-adherence among diabetes and hypertensive patients in Ghana: A qualitative investigation. PLoS One. 2018;13(3):e0193995. doi: 10.1371/journal.pone.0193995 29590156 PMC5874015

[24] Odusola AO, Hendriks M, Schultsz C, Bolarinwa OA, Akande T, Osibogun A, et al. Perceptions of inhibitors and facilitators for adhering to hypertension treatment among insured patients in rural Nigeria: a qualitative study. BMC Health Serv Res. 2014;14:624. doi: 10.1186/s12913-014-0624-z 25491509 PMC4267751

[25] Youssef RM, Moubarak II. Patterns and determinants of treatment compliance among hypertensive patients. East Mediterr Health J. 2002;8(4–5):579–92. doi: 10.26719/2002.8.4-5.579 15603041

[26] Mendoza JA, Lasco G, Renedo A, Palileo-Villanueva L, Seguin M, Palafox B, et al. (De)constructing “therapeutic itineraries” of hypertension care: A qualitative study in the Philippines. Soc Sci Med. 2022;300:114570. doi: 10.1016/j.socscimed.2021.114570 34802782 PMC7613024

[27] Nayeri ND, Dehghan M, Iranmanesh S. Being as an iceberg: hypertensive treatment adherence experiences in southeast of Iran. Glob Health Action. 2015;8:28814. doi: 10.3402/gha.v8.28814 26395925 PMC4579246

[28] Palafox B, Seguin ML, McKee M, Dans AL, Yusoff K, Candari CJ, et al. Responsive and Equitable Health Systems-Partnership on Non-Communicable Diseases (RESPOND) study: a mixed-methods, longitudinal, observational study on treatment seeking for hypertension in Malaysia and the Philippines. BMJ Open. 2018;8(7):e024000. doi: 10.1136/bmjopen-2018-024000 30061449 PMC6067392

[29] Government of the Philippines. Republic of the Philippines. Pantawid Pamilyang Pilipino Program (4P) Manila 2017 [cited 2025 12th May]. Available from: https://www.officialgazette.gov.ph/programs/conditional-cash-transfer/.

[30] Palafox B, Balabanova D, Loreche AM, Mat-Nasir N, Ariffin F, Md-Yasin M, et al. Pathways to Hypertension Control: Unfinished Journeys of Low-Income Individuals in Malaysia and the Philippines. Int J Health Plann Manage. 2025;40(2):442–57. doi: 10.1002/hpm.3889 39731689 PMC11897856

[31] Gee ME, Campbell N, Sarrafzadegan N, Jafar T, Khalsa TK, Mangat B, et al. Standards for the uniform reporting of hypertension in adults using population survey data: recommendations from the World Hypertension League Expert Committee. J Clin Hypertens (Greenwich). 2014;16(11):773–81. doi: 10.1111/jch.12387 25157607 PMC8031637

[32] USAID. The Demographic and Health Surveys(DHS) Program: USAID; 2022 [cited 2025 12th May]. Available from: https://dhsprogram.com/.

[33] World Health Organization. WHO STEPS Surveillance Manual: The WHO STEPwise approach to chronic disease risk factor surveillance/Non communicable diseases and mental health, World Health Organization Geneva: WHO; 2005.

[34] Nglehart RPB, Pettersson T. The world values survey. 2005. Available from: https://www.worldvaluessurvey.org/WVSDocumentationWV5.jsp

[35] Balabanova D, McKee M, Pomerleau J, Rose R, Haerpfer C. Health service utilization in the former soviet union: evidence from eight countries. Health Serv Res. 2004;39(6 Pt 2):1927–50. doi: 10.1111/j.1475-6773.2004.00326.x 15544638 PMC1361106

[36] Balabanova D, Roberts B, Richardson E, Haerpfer C, McKee M. Health care reform in the former Soviet Union: beyond the transition. Health Serv Res. 2012;47(2):840–64. doi: 10.1111/j.1475-6773.2011.01323.x 22092004 PMC3419892

[37] Filmer D, Pritchett LH. Estimating wealth effects without expenditure data--or tears: an application to educational enrollments in states of India. Demography. 2001;38(1):115–32. doi: 10.1353/dem.2001.0003 11227840

[38] Bovet P, Gervasoni J-P, Mkamba M, Balampama M, Lengeler C, Paccaud F. Low utilization of health care services following screening for hypertension in Dar es Salaam (Tanzania): a prospective population-based study. BMC Public Health. 2008;8:407. doi: 10.1186/1471-2458-8-407 19087300 PMC2615777

[39] Kotwani P, Balzer L, Kwarisiima D, Clark TD, Kabami J, Byonanebye D, et al. Evaluating linkage to care for hypertension after community-based screening in rural Uganda. Trop Med Int Health. 2014;19(4):459–68. doi: 10.1111/tmi.12273 24495307 PMC4118739

[40] Chung VQ, Morley K, O’Neil E, Ken N, Morley M. Evaluation of a hypertension screening programme in Independence, Belize. West Indian Med J. 2005;54(2):130–4. doi: 10.1590/s0043-31442005000200009 15999884

[41] Mekonnen HS, Gebrie MH, Eyasu KH, Gelagay AA. Drug adherence for antihypertensive medications and its determinants among adult hypertensive patients attending in chronic clinics of referral hospitals in Northwest Ethiopia. BMC Pharmacol Toxicol. 2017;18(1):27. doi: 10.1186/s40360-017-0134-9 28381241 PMC5382443

[42] Institute for Public Health. National health and morbidity survey 2015 (NHMS 2015). Vol. IV: Traditional and complementary medicine. Kuala Lumpur: Ministry of Health, Malaysia; 2015.

[43] Palileo-Villanueva LM, Palafox B, Amit AML, Pepito VCF, Ab-Majid F, Ariffin F, et al. Prevalence, determinants and outcomes of traditional, complementary and alternative medicine use for hypertension among low-income households in Malaysia and the Philippines. BMC Complement Med Ther. 2022;22(1):252. doi: 10.1186/s12906-022-03730-x 36180884 PMC9526286

[44] Macquart de Terline D, Kane A, Kramoh KE, Ali Toure I, Mipinda JB, Diop IB, et al. Factors associated with poor adherence to medication among hypertensive patients in twelve low and middle income Sub-Saharan countries. PLoS One. 2019;14(7):e0219266. doi: 10.1371/journal.pone.0219266 31291293 PMC6619761

[45] Perera M, de Silva CK, Tavajoh S, Kasturiratne A, Luke NV, Ediriweera DS, et al. Patient perspectives on hypertension management in health system of Sri Lanka: a qualitative study. BMJ Open. 2019;9(10):e031773. doi: 10.1136/bmjopen-2019-031773 31594895 PMC6797394

[46] Nashilongo MM, Singu B, Kalemeera F, Mubita M, Naikaku E, Baker A, et al. Assessing Adherence to Antihypertensive Therapy in Primary Health Care in Namibia: Findings and Implications. Cardiovasc Drugs Ther. 2017;31(5–6):565–78. doi: 10.1007/s10557-017-6756-8 29032396 PMC5730630

[47] Mukherjee JS, Ivers L, Leandre F, Farmer P, Behforouz H. Antiretroviral therapy in resource-poor settings. Decreasing barriers to access and promoting adherence. J Acquir Immune Defic Syndr. 2006;43 Suppl 1:S123-6. doi: 10.1097/01.qai.0000248348.25630.74 17133195

[48] Rogers JH, Jabateh L, Beste J, Wagenaar BH, McBain R, Palazuelos D, et al. Impact of community-based adherence support on treatment outcomes for tuberculosis, leprosy and HIV/AIDS-infected individuals in post-Ebola Liberia. Glob Health Action. 2018;11(1):1522150. doi: 10.1080/16549716.2018.1522150 30270812 PMC7012017

