# Supplementary material for: Study on health seeking behaviour and determinants of undiagnosed hypertension in poor households in the Philippines, part of the RESPOND study (SHARP-RESPOND)
Source: PLOS Glob Public Health. 2025 May 28;5(5):e0004550. doi: 10.1371/journal.pgph.0004550 (PMC12118906; doi:10.1371/journal.pgph.0004550)
Supplement: S1 Tables — (DOCX) [file pgph.0004550.s001.docx]

**Table A: List of household assets/characteristics used in the construction of the household wealth index using principal components analysis (mean and standard deviation)**

| **Household asset/characteristic** | **Mean** | **SD** | **N** |
| --- | --- | --- | --- |
| Motorcycle | 0.285 | 0.452 | 610 |
| Automobile | 0.049 | 0.216 | 610 |
| Bicycle | 0.267 | 0.443 | 610 |
| Tractor | 0.003 | 0.057 | 610 |
| Personal computer | 0.098 | 0.298 | 610 |
| Radio | 0.472 | 0.500 | 610 |
| Television | 0.884 | 0.321 | 610 |
| Telephone - landline | 0.025 | 0.155 | 610 |
| Telephone - mobile | 0.687 | 0.464 | 610 |
| Refrigerator | 0.498 | 0.500 | 610 |
| Washing machine | 0.462 | 0.499 | 610 |
| Food mixer | 0.010 | 0.099 | 610 |
| Air conditioner | 0.054 | 0.226 | 610 |
| Electric fan | 0.913 | 0.282 | 610 |
| Generator | 0.008 | 0.090 | 610 |
| Vacuum | 0.008 | 0.090 | 610 |
| Iron | 0.467 | 0.499 | 610 |
| Internet | 0.116 | 0.321 | 610 |
| Livestock - cattle | 0.120 | 0.325 | 610 |
| Livestock - poultry | 0.187 | 0.390 | 610 |
| Household cooking location - outside the house, covered | 0.079 | 0.269 | 610 |
| Household cooking location - outside the house, open | 0.054 | 0.226 | 610 |
| Household cooking location - partially inside the house | 0.041 | 0.198 | 610 |
| Household cooking location - completely inside the house | 0.826 | 0.379 | 610 |
| Primary cooking fuel - wood/agriculture/crop/other | 0.023 | 0.150 | 610 |
| Primary cooking fuel - charcoal/coal | 0.341 | 0.474 | 610 |
| Primary cooking fuel - kerosene/gas/electricity | 0.636 | 0.482 | 610 |

## Table B: Variable definition

| **Individual and household characteristics** | | **Exact questions & responses in survey** |
| --- | --- | --- |
| **Sex** | Male versus female sex | Sex – Female/Male |
| **Age** | Current age in years | Age in years |
| **Post secondary education** | Percentage of respondants completed secondary education or higher | What is your highest level of formal education achieved   - None/primary/secondary/vocational/college |
| **Married / cohabitating** | Percentage of married or living with partner | Marital status   - Never married/ married/ living with partner but not married/ widowed/ separated/ Divorced |
| **Currently employed** | No versus yes | Are you currently employed?   - Yes/No/ Refused |
| **Area of residence** | Rural versus Urban | Urban-rural setting   - Urban/ rural |
| **Asset-based wealth score** | 0-1, 1 being wealthiest |  |
| **Household size** | Number of household members | What is the number of individuals living in your household? |
| **Monthly income** | Total household monthly income, in Philippine Peso (PH Peso) | What is your current total monthly household income in Pesos after deducting taxes (including employment, pensions, social assistance, family support, from family abroad, etc.) |
| **Total monthly household expenditure per capita** | Total monthly household expenditure per capita, PH Pesos | How much was spent in the last 30 days on the followings: healthcare, rent, clothing, transportation |
| **Logged monthly household health expenditure per capita** | Monthly household health expenditure per capita in log scale, PH Peso | / |
| **Total monthly household health expenditure** | Total monthly household expenditure on health, PH Peso | / |
| **Self-rated poor** | Self-rated on overall financial situation to be very poor/poor versus neither good nor bad/good/very good | As a whole, how would you assess the financial situation of your household in the last month?  Very good, good, neither good nor bad, poor, very poor |
| **Presence of NCD comorbidity** | Presence of non-communicable disease as a comorbidity  No versus Yes | Have you been diagnosed with a non-communicable disease – diabetes, cancer, congestive heart failure, heart attack, others |
| **Self-rated poor health** | Self -rated on overall health to be very poor/poor versus neither good nor bad/good/very good | As a whole, how would you describe your health?   - very poor/poor versus neither good nor bad/good/very good |
| **Receipt of any health information in the past 12 months** | No versus yes | In the past 12 months, have you received/ heard/ seen any information or messages about high blood pressure or how to prevent diseases that affect the heart?   - Yes/No/Refused |
| **Blood pressure at first visit** | Average of the 3 readings of systolic and diastolic blood pressure taken during first visit | / |
| **Blood pressure at follow up** | Average of the 3 readings of systolic and diastolic blood pressure taken during follow up | / |
| **Regular monthly blood pressure monitoring at least monthly** | Self-reported having blood pressure measured once every 2-3 years or more seldom/yearly/several times a year versus several times a month/several times a week/daily | Overall, how often is your blood pressure measured currently?   - Daily,several times a week, several times a month, several times a year, once a year, once every 2-3 years or more seldom, don’t know, refused |
| **Knowledge on hypertension and health perception** | |  |
| **Self-rated to have poor knowledge on hypertension** | Self-rated knowledge level to know nothing at all/have only heard of the term versus know a little/am very familiar with hypertension | How much do you know about ‘blood pressure’?   - Nothing at all, I have only heard the term, I know a little about it, I am very familiar with it, refused |
| **Score on knowledge of hypertension (out of 5)** | A score out of 5 computed from correct responses to 5 questions on hypertension – the higher the score, the more knowledgeable | A score of 5 out of responding correctly to whether they agree with the following questions, each correct answer equates to 1 point. (Yes/No/don’t know, refused)   1. High blood pressure can cause a stroke 2. High blood pressure can cause cancer 3. People with high blood pressure generally feel well and do not notice that they have high blood pressure 4. People with high blood pressure can stop taking their medications when their blood pressure value is normal 5. People with high blood pressure only have to take their medication when they feel unwell |
| **Score for fatalistic view** | A score out of 4 computed from responses to 4 questions on fatalistic view on health – the higher the score, the more fatalistic | 1 score for agree/strongly agree to each of the statements. (Strongly agree, agree, disagree, strongly disagree, don’t know, refused)   1. There is nothing you can do; health is determined by heredity 2. Keeping healthy depends upon the things that one can do 3. There are certain things that one can do for oneself to reduce the risk of heart attack 4. There are certain things that one can do for oneself to reduce the risk of getting cancer |
| **All things considered, how satisfied are you with your life** | A score out of 10 on satisfaction with life, 10 being most satisfied | All things considered, how satisfied are you with your life as a whole these days? If 1 means you are “very dissatisfied” on the scale below and 10 means you are “very satisfied”, where would you put your satisfaction with your life as a whole?   - 1(very dissatisfied) – 10 (very satisfied); refused |
| **Do you feel they have completely free choice and control over your life?** | A score out of 10 on perceived control over life | Some people feel they have completely free choice and control over their lives, while other people feel that what they do has no real effect on what happens to them. Please use this scale where 1 means “no choice at all” and 10 means “a great deal of choice” to indicate how much freedom of choice and control you feel you have over the way your life turns out.   - 1(no choice at all) – 10(great deal of choice); refused |
| **Believes that western medicines are effective** | Believe that western medications are very effective/effective versus sometimes effective/ineffective/very ineffective | Please rate western medicines as not effective, effective or very effective to reduce blood pressure. |
| **Believes that traditional medicines are effective** | Believe that traditional medications are very effective/effective versus sometimes effective/ineffective/very ineffective | Please rate traditional medicines as not effective, effective or very effective to reduce blood pressure. |
| **Social participation and cohesion** | |  |
| **Active member of sports or recreational organization** | Self-reported to be an active member versus don’t belong/inactive member of sports or recreational organization | Are you actively involved in sport or recreational organisations? |
| **Active member of art, music or educational organizations** | Self-reported to be an active member versus don’t belong/inactive member of art, music or educational organizations | Are you actively involved in Art, music or educational organisations? |
| **Active member of humanitarian or charitable organizations** | Self-reported to be an active member versus don’t belong/inactive member of humanitarian or charitable organizations | Are you actively involved in humanitarian or charitable organisations? |
| **Active member of church/religious organizations** | Self-reported to be an active member versus don’t belong/inactive member of church/religious organizations | Are you actively involved in church or religious organisations? |
| **Trust score personal** | A score out of 5 for having expressed trust (answered Quite a lot/ A great deal versus Not at all/ not very much)for each of the 5 groups of people. | Tell me for each whether or not you trust people from each of these groups:   - Your neighbourhood - People you know personally - People you meet for the first time - People of another religion - People of another nationality |
| **Have trust in the police** | Self-reported confidence level in police not at all/not very much versus quite a lot/a great deal | How much confidence do you have in the following organisations? The police |
| **Have trust in the court** | Self-reported confidence level in court not at all/not very much versus quite a lot/a great deal | How much confidence do you have in the following organisations? The courts |
| **Have trust in the government** | Self-reported confidence level in the government not at all/not very much versus quite a lot/a great deal | How much confidence do you have in the following organisations? The government |
| **Have trust in the health system** | Self-reported confidence level in the health system not at all/not very much versus quite a lot/a great deal | How much confidence do you have in the following organisations? The health system |
| **Score for trust in public institutions (out of 4)** | A score out of 4 computed from responses of trust towards the police, the court, the government and the health system – the higher, the more trust | A score out of 4. 1 score for answering quite a lot/ a great deal for each of the above 4 institutions – The police, the court, the government and the health system |

## Table C: Medical adherence and health seeking behaviour of previously known, newly diagnosed and undiagnosed hypertensives.

|  | | **Previously diagnosed hypertensives at Baseline(n=379)** | **Previously diagnosed hypertensives at FU(n=379)** | **Undiagnosed hypertension at baseline (n=137)** | **Undiagnosed hypertension at baseline but received formal diagnosis at FU (n=33)** | **Undiagnosed hypertension with no formal diagnosis (n=97)** |
| --- | --- | --- | --- | --- | --- | --- |
| **Currently taking medication for hypertension** | No | 16.2% ± 0.5% | 11.1% ± 0.3% | / | 45.4% ± 0.6% | / |
|  | Yes - Same med as when diagnosed | 52.4% ± 0.4% | 80.6% ± 0.4% | / | 54.0% ± 0.9% | / |
|  | Yes - Medication changed since diagnosis | 31.4% ± 0.5% | 8.4% ± 0.0% | / | 0.6% ± 0.6% | / |
| **Consistently take medication for hypertension in the past 12 months** |  | 66.2% ± 0.8% | 41.3% ± 0.5% | / | 23.5% ± 1.1% | / |
| **How often do you forget to take medication?** | Never | 30.8% ± 0.6% | 37.6% ± 0.8% | / | 38.9% ± 4.4% | / |
|  | Some of the time | 59.8% ± 0.4% | 61.3% ± 0.9% | / | 61.1% ± 4.4% | / |
|  | Most of the time | 3.6% ± 0.2% | 1.0% ± 0.2% | / | 0.0% ± 0.0% | / |
|  | All the time | 5.7% ± 0.3% | 0.0% ± 0.0% | / | 0.0% ± 0.0% | / |
| **How often do you decide not to take medication?** | Never | 46.0% ± 0.4% | 68.4% ± 0.6% | / | 70.5% ± 2.2% | / |
|  | Some of the time | 23.9% ± 0.6% | 27.0% ± 0.5% | / | 26.9% ± 1.3% | / |
|  | Most of the time | 21.1% ± 0.2% | 10(4.9%) | / | 2.7% ± 2.6% | / |
|  | All the time | 8.9% ± 0.3% | 1(0.5%) | / | 0.0% ± 0.0% | / |
| **How often do you miss your medications because you feel better?** | Never | 37.9% ± 0.1% | 68.8% ± 0.8% | / | 83.7% ± 19% | / |
|  | Some of the time | 31.6% ± 0.3% | 28.1% ± 0.6% | / | 16.3% ± 1.9% | / |
|  | Most of the time | 19.3% ± 0.5% | 2.3% ± 0.2% | / | 0.0% ± 0.0% | / |
|  | All the time | 11.2% ± 0.6% | 0.9% ± 0.0% | / | 0.0% ± 0.0% | / |
| **How often do you decide to take less of your medicine?** | Never | 78.8% ± 0.4% | 98.8% ± 0.2% | / | 100.0% ± 0.0% | / |
|  | Some of the time | 13.4% ± 0.3% | 1.2% ± 0.2% | / | 0.0% ± 0.0% | / |
|  | Most of the time | 6.1% ± 0.1% | 0(0.0%) | / | 0.0% ± 0.0% | / |
|  | All the time | 1.7% ± 0.1% | 0(0.0%) | / | 0.0% ± 0.0% | / |
| **How often do you stop your medication because you feel sick from the side effects?** | Never | 79.4% ± 0.4% | 99.9% ± 0.1% | / | 100.0% ± 0.0% | / |
|  | Some of the time | 12.2% ± 0.3% | 0.1% ± 0.1% | / | 0.0% ± 0.0% | / |
|  | Most of the time | 8.4% ± 0.3% | 0.0% ± 0.0% | / | 0.0% ± 0.0% | / |
|  | All the time | 0.0% ± 0.0% | 0.0% ± 0.0% | / | 0.0% ± 0.0% | / |
| **How often do you forget to bring along your medication during travel?** | Never | 77.1% ± 0.6% | 99.9% ± 0.1% | / | 86.0% ± 0.7% | / |
|  | Some of the time | 18.5% ± 0.5% | 0.1% ± 0.1% | / | 14.0% ± 0.7% | / |
|  | Most of the time | 1.8% ± 0.1% | 0.0% ± 0.0% | / | 0.0% ± 0.0% | / |
|  | All the time | 2.6% ± 0.3% | 0.0% ± 0.0% | / | 0.0% ± 0.0% | / |
| **How often do you not take your medication because you ran out of them at home?** | Never | 56.4% ± 0.6% | 71.2% ± 1.2% | / | 91.7% ± 1.9% | / |
|  | Some of the time | 28.3% ± 0.7% | 26.8% ± 1.3% | / | 6.8% ± 2.5% | / |
|  | Most of the time | 11.5% ± 0.1% | 2.0% ± 0.4% | / | 1.5% ± 1.5% | / |
|  | All the time | 3.8% ± 0.2% | 0.0% ± 0.0% | / | 0.0% ± 0.0% | / |
| **Who is your main health provider for hypertension treatment?*** | Hospital | 14.9% ± 0.2% | 0.5% ± 0.1% | 12.4% ± 2.2% | 0.0% ± 0.0% | 0.3% ± 0.3% |
|  | Clinic | 52.0% ± 0.2% | 93.9% ± 0.2% | 44.3% ± 1.4% | 86.5% ± 0.3% | 68.6% ± 1.1% |
|  | Pharmacist at retail pharmacy | 0.0% ± 0.0% | 4.2% ± 0.1% | 0(0.0%) | 15.2% ± 0.3% | 13.1% ± 0.7% |
|  | Private physician | 2.9% ± 0.1% | 0.0% ± 0.0% | 1.6% ± 0.2% | 0.0% ± 0.0% | 0.0% ± 0.0% |
|  | Community health workers | 1.0% ± 0.1% | 0.0% ± 0.0% | 0.5% ± 0.2% | 0.0% ± 0.0% | 0.0% ± 0.0% |
|  | Traditional healer | 0.0% ± 0.0% | 0.2% ± 0.2% | 0.9% ± 0.7% | 0.0% ± 0.0% | 1.6% ± 1.0% |
|  | Friends/Family/Colleagues | 17.7% ± 0.3% | 0(0.0%) | 19.6% ± 0.2% | 0.0% ± 0.0% | 0.0% ± 0.0% |
|  | Others | 3.4% ± 0.1% | 0.9% ± 0.2% | 10.5% ± 0.6% | 0.0% ± 0.0% | 16.4% ± 0.9% |
|  | Don’t know | 8.1% ± 0.1% | 0(0.0%) | 10.1% ± 0.7% | 0.0% ± 0.0% | 0.0% ± 0.0% |
| **How often do you see these providers** | Less than once per year | 19.9% ± 0.3% | 33.8% ± 0.2% | 34.0% ± 1.0% | 30.0% ± 1.1% | 79.0% ± 1.7% |
|  | Once every 6-12 months | 12.3% ± 0.3% | 13.9% ± 0.3% | 16.8% ± 0.9% | 34.8% ± 0.9% | 4.0% ± 0.8% |
|  | Once every <6 months | 26.4% ± 0.2% | 18.4% ± 0.2% | 15.1% ± 0.2% | 12.2% ± 0.3% | 5.7% ± 0.3% |
|  | Monthly | 28.8% ± 0.3% | 33.4% ± 0.2% | 12.8% ± 0.8% | 23.0% ± 1.3% | 1.6% ± 0.7% |
|  | Don’t know | 12.7% ± 0.2% | 0.4% ± 0.0% | 21.3% ± 1.3% | 0.0% ± 0.0% | 9.7% ± 0.6% |
| **How often is your blood pressure measured?** | Once every 2-3 years or less frequent | 1.4% ± 0.0% | 1.0% ± 0.1% | 21.5% ± 0.8% | 0.0% ± 0.0% | 16.1% ± 1.1% |
|  | Yearly | 1.7% ± 0.2% | 4.3% ± 0.2% | 6.3% ± 0.8% | 6.8% ± 0.9% | 13.9% ± 1.3% |
|  | Several times a year | 23.6% ± 0.4% | 36.9% ± 0.4% | 32.0% ± 0.6% | 50.1% ± 1.5% | 54.7% ± 1.7% |
|  | Monthly or more frequent | 73.3% ± 0.4% | 57.8% ± 0.2% | 35.7% ± 0.1% | 24.2% ± 0.7% | 15.2% ± 0.8% |
|  | Don’t know | 0.0% ± 0.0% | 0.0% ± 0.0% | 4.6% ± 0.2% | 18.9% ± 0.0% | 0.0% ± 0.0% |
| **Have easy access to blood pressure measuring device** |  | / | 94.4% ± 0.4% | / | 82.7% ± 0.9% | 78.0% ± 4.2% |

##

## Table D: Derivation of the probability-based sampling weights

Sampling weights are needed to analyse the household survey data to avoid biased estimates due to the multi-stage sampling approach that selected communities in each rural and urban strata with probability proportional to their varying population size, an equal number of households within each community, and one household member to participate among all those eligible within the household. Individual-level weights are calculated by taking the inverse product of the:

- Unconditional probability of selecting the community (mukim/barangay) within the state/province (e.g. mukim population/state population at the time of data collection)
- Conditional probability of selecting household within the community (e.g. number of households screened in mukim/total number of households in mukim)
- Conditional probability of selecting participant from all eligible residents within household (i.e. 1/number of eligible adults screened within the household)
